# Comprehension priming in Chinese EFL learners’ reflexive pronoun interpretation

**DOI:** 10.3389/fpsyg.2024.1425061

**Published:** 2024-08-30

**Authors:** Lianrui Yang, Zimiao Song, Yinxia Wei

**Affiliations:** College of Foreign Languages, Ocean University of China, Qingdao, China

**Keywords:** comprehension priming, Chinese EFL learners, binding theory, English reflexive pronouns, locality constraint

## Abstract

Reflexive interpretation is a pivotal aspect of discourse comprehension, which usually reveals consistent challenges for Chinese EFL learners. These learners often breach the locality constraint of reflexive pronouns, exhibiting a persistent tendency towards optional reflexive comprehension. Recent research has demonstrated the effectiveness of the priming technique in altering biases among L2 learners in anaphora resolution. However, no existing studies have investigated comprehension priming in the context of reflexive interpretation among Chinese EFL learners. This study addresses this gap by conducting a sentence comprehension experiment with 36 high school students to explore the potential of comprehension priming in modifying L2 learners’ reflexive interpretation biases and examining the persistence of the priming effect. The findings reveal immediate and cumulative priming effects, with no discernible effect observed after 1 week. The results suggest that comprehension priming can occur universally, even without lexical overlap, previously assumed to be a prerequisite. While the priming effect lacks statistical significance after 1 week, there is a numerical increase in participants consistently interpreting the target structure correctly. Thus, comprehension priming emerges as an effective method for L2 learners to internalize more abstract linguistic rules. Further research on comprehension priming across diverse L2 populations and language structures is warranted.

## 1 Introduction

Reflexive interpretation has long been studied because of its important role in discourse comprehension, and it has posed consistent difficulty for learners of English as a foreign language (EFL). Most previous studies on EFL learners’ reflexive comprehension have focused on the differences between EFL learners and native speakers in comprehending reflexive pronouns in different sentences, and what factors affect their comprehension. EFL have difficulty acquiring the binding properties such as locality, orientation, and c-command, with locality as a more studied topic (Wu et al., 2020). Therefore, this article also puts an emphasis on the binding property of locality. There is an agreement that the use of reflexive pronouns, unlike pronouns, involves parametric diversity and, as a result, reflexives are bound differently in different languages (Wexler and Manzini, 1987). As stipulated by Chomsky’s binding principle A, the reflexive should be bound within its governing category. The governing category for English reflexives is the minimal category which contains the reflexive and a subject, thus English reflexives can only be bound locally. The governing category for Chinese reflexive *ziji* is the minimal category which contains *ziji* and a root tense, thus allowing both local and long-distance bindings. Therefore, Chinese EFL learners may overextend the overlapping uses of Chinese *ziji* in English reflexive interpretation. It has been demonstrated that Chinese EFL learners’ reflexive interpretation may still be influenced by their L1 even at a high proficiency level (Wang, 2000). Although EFL learners are proven to be more likely to bind reflexive pronouns as their English proficiency improves or when the sentence has a semantic or pragmatic preference denoting that the reflexive pronoun is co-referential with the local NP, few studies have explored how to help L2 learners change their initial interpretation biases or acquire the binding property in a relatively short time.

Structural priming, also called syntactic persistence or syntactic priming, is the facilitation of the processing of a sentence preceded by another sentence that has the same syntactic structure without one’s conscious intent or awareness. Even though structural priming has been commonly found in language production (Levelt and Kelter, 1982; Bock, 1986; Bock and Griffin, 2000), priming in comprehension has been less studied, and the results are more varied. Some researchers believe that comprehension priming has a dominant dependency on lexical overlap (Raissi et al., 2020; Tooley and Traxler, 2010). However, there is evidence that lexical overlap is not the prerequisite for comprehension priming (Luka and Barsalou, 2005; Ledoux et al., 2007; Viau et al., 2010; Contemori, 2021; Contemori et al., 2022). The comprehension priming effect has been found for various syntactic structures, for example, alternative structures such as double-object vs. prepositional-object constructions and active vs. passive sentences. Only recently has priming in anaphora resolution begun to receive some attention. To get a better understanding of comprehension priming, more studies with different L2 populations and language structures are needed.

The present study explored comprehension priming in reflexive interpretation for Chinese EFL learners. It is a partial replication of two studies conducted by Contemori (2021) and Contemori et al. (2022) as a response to the advocacy for more similar studies with different bilingual populations and language structures. Two specific objectives include: firstly, to test whether earlier access to local antecedent interpretation in prime sentences can facilitate similar comprehension in subsequent experimental sentences; and secondly, to explore the persistence of the priming effects by examining L2 learners’ reflexive interpretation immediately after the presentation of the priming sentence, after the whole priming phase, and after 1 week.

## 2 Literature review

### 2.1 Reflexive pronoun interpretation for EFL learners

#### 2.1.1 English reflexive pronouns and the binding theory

Reflexive pronoun is a form of a pronoun that is used when the direct or indirect object in a sentence refers to the same person or thing as the subject of the sentence. Reflexive pronouns “reflect” or get their meaning from another nominal element or NP of the clause, usually the subject. The NP serves as the antecedent of the reflexive pronoun, and they are in a coreferential relation (Quirk et al., 1985). One of the most important theories dealing with indexing relationships between nominal expressions is Binding Theory by Chomsky. Following Principle A, a reflexive pronoun must be bound within its governing category or binding domain and it can only be co-indexed with the NP within the binding domain. For example, in sentence (1), the binding domain of *herself* is the subordinate clause, and *herself* can only refer to the local NP *Mary* instead of the distant NP *Susan* which is a constituent of the main clause. Sentences that violate Principle A are ungrammatical because the locality constraint cannot be overridden by contextual information. Sentence (2) is ungrammatical for the lack of gender agreement between the reflexive pronoun and its antecedent.

1 Susan_i_ heard that [Mary_j_ had bought herself_j/i*_ a new bicycle].
2 *Susan heard that Mike had bought herself a new bicycle.

Principle A is not without problems because it fails to explain the binding properties of reflexive pronouns in different languages. Reflexive pronouns can be divided into two categories based on whether they obey the same anaphor-binding principle as English reflexive pronouns: unmarked reflexive pronouns and marked reflexive pronouns. English reflexive pronouns belong to the unmarked class of reflexives. In Chinese, there are two possible analogs to English reflexive pronouns: *ziji*, which can be seen as “self,” and a form inflected for person and number, such as *taziji* and *woziji*. The latter analog has the same grammatical nature as English reflexive pronouns and can be characterized as an unmarked reflexive pronoun. For example, in sentence (3), *taziji* can only refer to the local antecedent XiaoLi within the binding domain. The morphologically simpler reflexive *ziji*, however, can be seen as a marked reflexive pronoun because it has some language-specific restrictions. Chinese *ziji* can be anteceded by both local and long-distance subjects, as in sentence (4). In other words, the antecedent can appear in any position in the sentence that commands *ziji*.

3 小张_i_听说小李_j_骂他自己_j/i*_。

XiaoZhang_i_ tingshuo XiaoLi_j_ ma taziji_j/i*_.

XiaoZhang_i_ heard that XiaoLi_j_ swore at himself_j/i*_.

4 彼得_i_认为约翰_j_相信自己_i/j_。

Peter_i_ renwei John_j_ xiangxin ziji_i/j_.

Peter_i_ thinks that John_j_ trusts self_i/j_.

#### 2.1.2 Previous studies on EFL learners’ reflexive pronoun interpretation

Previous studies have explored factors that affect EFL learners’ reflexive pronoun interpretation, including contextual information, L2 proficiency, sentence type, and so on (Jackson, 2018). EFL learners are more susceptible to semantic or pragmatic factors in reflexive pronoun interpretation than native English speakers. Previous studies usually used experimental sentences that contained pragmatic preferences. Thomas (1989) demonstrated that there was a significant difference between EFL learners and native speakers in following reflexive pronouns’ local-binding requirement, especially in pragmatically unsupportive contexts. The author concluded that the difference between native speakers and EFL learners was that the latter frequently permitted a reflexive pronoun in a finite subordinate clause to be bound by a distant NP, whether it was pragmatically favored or not. Similar results were attained in Chen’s study (Chen, 2001). When the distant NP was pragmatically favored, all responses of native speakers were correct while the comprehension accuracy was only 45% even for the most proficient group of EFL learners.

Although the local-binding rule is difficult to acquire for EFL learners, they show a tendency to bind the reflexive pronoun locally as their English proficiency improves. Studies have proven that learners’ anaphora resolution improves with the development of their L2 proficiency (Chen, 2001; Li, 2002; Wu, 2014, 2017). Chen (2001) tested the interpretation of English reflexive pronouns among three groups of Chinese EFL learners with varying proficiency levels. Additionally, a control group consisting of native speakers was recruited for comparison. The results showed that, in general, the higher the participants’ English proficiency, the higher the comprehension accuracy. However, even the group with the highest proficiency level performed worse than the control group, suggesting that EFL learners had difficulty acquiring the binding rules.

Another factor that may affect EFL learners’ reflexive pronoun interpretation is sentence type. A common finding is that learners are more likely to follow the local-binding requirement when comprehending reflexive pronouns in bi-clausal finite sentences than in bi-clausal nonfinite sentences (Finer and Broselow, 1986; Hirakawa, 1990; Chen, 2001; Wang, 2000). Matsumura (2007) refers to this phenomenon as “tensed-infinitive asymmetry.” In fact, White et al. (1997) found that the mean score of bi-clausal infinite sentences was a little lower than that of bi-clausal finite sentences even for the native speakers.

### 2.2 Structural priming and comprehension priming

Structural priming occurs when processing of a target sentence is facilitated following processing of a prime sentence that has the same syntactic structure (Bock, 1986). It has been experimentally demonstrated that repeated syntactic structure facilitates both production and comprehension, though studies on the latter are fewer in number and have mixed results. Structural priming in L2 comprehension, or comprehension priming, is defined by Contemori et al. (2022, p. 5) as “the technique where repeated exposure to a particular structure can facilitate the online and off-line comprehension of a following structure of the same type.” Three major theories have been proposed to explain the underlying mechanisms of structural priming in language production and comprehension: a residual activation account (Pickering and Branigan, 1998), an implicit learning account (Bock and Griffin, 2000), and a dual mechanism account (Tooley and Traxler, 2010). Residual activation mechanism views structural priming as short-term activation at the lemma stratum. The word involves activation of lemmas, feature nodes, and combinatorial nodes. Implicit learning mechanism views structural priming as a form of long-term implicit learning that does not require explicit memory for the form of the prime (Kaan and Chun, 2018). Repeated exposure to a particular structure strengthens the connections between the elements of that structure. Based on the above two accounts, the dual mechanism is proposed. According to this model, priming effects with different time courses are caused by different mechanisms. Lexically-independent syntactic priming effects are caused by an implicit learning mechanism, whereas lexically dependent priming effects are caused by a more short-term learning mechanism (Tooley and Traxler, 2010). Structural priming was first found in language production by Levelt and Kelter (1982) in native Dutch speakers. Using a question-answering format, they found that a question’s surface form can affect the format of the answer. However, the observed priming effect diminished when there was additional verbal material following the prime question. Bock and Griffin (2000) investigated priming over lags and found that the lexically-independent priming effect could persist over ten intervening sentences. Later studies have found that priming in production can occur in the absence of lexical overlap, which supports the existence of abstract syntactic representations (Bock, 1986), though the magnitude of the priming effect indeed increases when the prime and the target sentences share the same content words, an effect called “lexical boost.”

Studies on comprehension priming have had various results, and comprehension priming effect is frequently observed when there is verb repetition between the prime and target sentences (Raissi et al., 2020; Tooley and Traxler, 2010). Arai et al. (2007) believed that priming during comprehension is completely lexically dependent. The materials of their study were dative (DO/PO) alternations and the participants were native English speakers. The eye-movement data showed that the priming effect was observed only when the prime sentence and the target sentence shared the same verb. Other studies, however, show that comprehension priming can occur without lexical repetition. In a different paradigm, Luka and Barsalou (2005) also recruited native English speakers as participants who were required to read English sentences and later rate identical, structurally similar, or novel sentences for grammatical acceptability. No content word was repeated between sentences in the preceding reading list and corresponding sentences in the later rating list. The results showed that participants’ ratings were significantly higher if they had been exposed to identical or structurally similar sentences during the reading task even after a five-minute distractor task. Another study conducted by Ledoux et al. (2007) with native English speakers further verified Luka and Barsalou’s conclusion with an ERP experiment. The participants read target sentences containing reduced-relative (RR) clauses that were preceded by sentences that contained either the same RR clauses or main-clause (MC) constructions. The results implied that the semantic facilitation effect or priming effect was dissociated from effects of verb repetition.

In the field of second language acquisition, studies on comprehension priming also have mixed results. In the study of Kaan et al. (2019), both native English speakers and Spanish English learners were recruited for a self-paced reading task. The purpose was to examine whether exposure to temporarily ambiguous structures could change participants’ future expectations and reduce the reading time of sentences with the same structure. It was found that only native speakers showed an adaptation for only one of the two studied structures, implying that adaptation might be determined by many factors, such as proficiency, structure type, and task-related features. Two studies conducted by Contemori (2021) and Contemori et al. (2022) delved into the interpretation of pronouns by Spanish EFL learners within varied discourse structures. These studies revealed that comprehension priming effectively altered participants’ biases in pronoun interpretation. Notably, the priming effect endured over an extended period of 6–10 days (Contemori et al., 2022). The findings of these investigations suggest that the comprehension priming effect stemmed not from lexical cues, but rather from the implicit learning of specific patterns.

In the present study, reflexive pronouns’ locality constraint is chosen as the learning target because this property plays an important role in reflexive pronoun interpretation, and previous priming studies have seldom investigated comprehension priming in Chinese EFL learners. This priming study will help to find ways that may enhance the acquisition of reflexive pronouns’ locality property and provide suggestions for teachers to enhance the design of pedagogical techniques.

### 2.3 Research questions

This study aims to investigate how Chinese EFL learners utilize evidence extracted from the input in reflexive interpretation. Two types of experimental sentences with varying pragmatic biases are created, including sentences with unsupportive pragmatic information to assess whether participants can acquire the true locality constraint. By integrating the findings from Contemori (2021) and Contemori et al. (2022), it aims to provide an updated perspective on reflexive interpretation and proposes the following two research questions:

Research question 1: Does comprehension priming occur in reflexive interpretation for Chinese EFL learners? If so, what priming effect does it produce?

Research question 2: How persistent are the priming effects in reflexive interpretation for Chinese EFL learners?

## 3 Methodology

### 3.1 Participants

Thirty-six Chinese EFL learners participated in the exposure or priming phase (16 males, 20 females; mean age = 16.9; SD = 0.4; range = 16–18). Informed consent was obtained from each participant, ensuring that they willingly agreed to take part in the research. All participants were recruited from the same senior high school class in Linyi, Shandong Province. Senior high school students represent a critical stage in language development where they are actively refining their language skills, particularly in an educational context that prepares them for higher education or professional careers. This stage is pivotal for understanding how learners at this developmental phase adapt to and internalize syntactic structures through priming effects. While many studies involve university students or adults, investigating high school students provides insights into a younger cohort with potentially different cognitive and linguistic processing capabilities. This study can contribute to a comparison that allows us to explore whether age or educational stage influences the susceptibility to structural priming effects in reflexive interpretation.

Adopting a selection criteria used in previous studies (Contemori et al., 2022), only participants who scored 60% or lower on the local antecedent interpretation in the pretest were included in the sample. It is believed that exposure can only be effective if participants’ reflexive interpretations are different from the nativelike pattern (Contemori et al., 2022).

The participants were in Grade 11, and their English proficiency was measured by a Quick Placement Test (QPT). Their scores were all between 24 and 39 (mean = 30.5 over 60; SD = 4.0); hence, they could be considered to be at the intermediate level (B1-B2) of the Common European Framework of Reference. The experiment was conducted by their teacher in two self-study classes. The participants were informed that their participation was voluntary and they could withdraw at any time.

Thirty-three participants who had participated in the first experiment session took part in the second session, which took place at the same self-study class after 1 week.

### 3.2 Materials

The target sentence pattern was the bi-clausal finite sentence. The subject of the matrix clause was referred to as NP1, as it appeared first in the sentence, or as the distant NP because of the longer distance between it and the reflexive. The subject of the subordinate clause, also the antecedent of the reflexive, was referred to as NP2 or local NP. An example is shown in (5). In the exposure phase, 30 experimental sentences were constructed. Two NPs were of the same gender as the reflexive.

5 David (NP1/distant NP) could see that Bill (NP2/local NP) was looking at himself in the mirror.

Although verbs in all subordinate clauses were different to test whether structural priming in comprehension could occur without verb repetition, verbs in matrix clauses did repeat occasionally, with efforts being made to ensure that matrix clauses of the same pair (the experimental sentence and the sentence preceding it) did not share the same verb. These experimental sentences were designed to contain as little semantic and pragmatic bias as possible to ensure that participants would choose the antecedent based on their own interpretation biases. Each experimental sentence was followed by a comprehension question to test reflexive interpretation. Three choices were given: one choice corresponded to NP1, one corresponded to NP2, and the third answer was consistently “either NP1 or NP2.” The positions of the first two choices in the multiple-choice question were counterbalanced across the experiment, while the third remained constant. An example is illustrated in (6). No feedback was provided to participants throughout the experiment.

6 David could see that Bill was looking at himself in the mirror. Who did Bill see in the mirror?

- Bill.
- David.
- Either Bill or David.

In the exposure phase, half of the experimental sentences were preceded by prime sentences, and the other half were preceded by baseline sentences.. Each experimental sentence was followed by a filler sentence. The baseline sentences did not contain any reflexives and were created in the same way as filler sentences, as illustrated in (7). The prime sentences, containing contextual information that made the NP2 a more plausible antecedent and reflexive interpretation easier, shared the same structure as the experimental sentences, but the two NPs differed in gender. An example of a prime sentence is illustrated in (8).

7 Sophie wrote a note for her parents before she went to a party with her boyfriend. Who was the note written for?
8 The mother is worried that her son would injure himself while skating on the streets. Who is the mother worried about?

The priming phase involved 90 sentences in total: 30 experimental sentences, 15 prime sentences, 15 baseline sentences, and 30 filler sentences. There were two versions of the exposure phase, such that an experimental sentence was preceded by a prime sentence in one list but by a baseline sentence in another list. The pretest, immediate posttest, and delayed posttest each contained 10 experimental sentences and five filler sentences. In each test, half of the experimental sentences were created in the same way as those in the exposure phase, but the other half were designed to contain a pragmatic bias that made the NP1 more plausible to test whether participants would be more likely to reject long-distance binding after the exposure phase. An example of such a biased experimental sentence is illustrated in (9).

9 Julie complained to me that her mother always compares herself with their neighbors. Who is being compared with the neighbors?

The neutral and biased experimental sentences were presented in a random order, with a filler sentence between every two sentences. The testing materials for the pretest and the delayed posttest were the same. The immediate posttest used another set of sentences that were equivalent in difficulty to those in the pretest.

The pretest, exposure phase, and immediate posttest were presented in one session by the course coordinator. Participants were unaware that the test consisted of three separate phases. After a week, they completed the delayed posttest and a quick placement test. The whole experiment contained 135 sentences, with 120 in the first three phases and 15 in the final session. The structure of the experiment is summarized as follows.

Table 1 Example of the structure of the task.

**Table 1:** Example of the structure of the task.

Pretest	10 experimental sentences and 5 filler sentences
Exposure phase	30 experimental sentences, 15 prime sentences, 15 baseline sentences, 30 filler sentences; Order of presentation: prime-experimental-filler-baseline-experimental-filler
Immediate posttest	10 experimental sentences and 5 filler sentences
Delayed posttest	10 experimental sentences and 5 filler sentences

### 3.3 Procedure and coding

The experiment was conducted by the head teacher in two sessions with the second one administered a week later. Each session took 45 min. Although there was no time limit for participants to finish the comprehension task, all participants finished within 45 min. The materials were printed and stapled into booklets with the intention of preventing participants from looking back and summarizing the explicit rule. Only one participant changed the original answers to several questions in succession during the experiment, and this data was discarded. The participants were not informed of the purpose of the test throughout the whole process. In the first session, participants were first asked for some basic information, such as their age, gender, and English learning duration (year). During the test, participants were allowed to use a dictionary to consult explanations of unknown words, though Chinese definitions had been annotated for some important words that, in their teacher’s view, might be unfamiliar to them. After the entire experiment, an online interview was given to five random participants to explore whether they had realized the purpose of the experiment and whether they had found a rule in reflexive interpretations. They all regarded the experiment as a reading comprehension test and could not summarize the explicit rule.

If a participant’s accuracy on all sentences except experimental sentences in the first session was lower than 80%, then his or her data would not be included in the subsequent analysis. This was to ensure that the materials of the study were well understood. The result showed that participants’ accuracy on these sentences was on average 91.4%, suggesting good comprehension of the materials. As for the experimental sentences, the local antecedent choice was coded 1, while the distant antecedent choice was coded 0. In the pretest, participants who correctly comprehended more than six out of 10 experimental sentences were thought to have a good command of the local-binding property, and their data would not be analyzed either in case of the ceiling effect.

## 4 Results

### 4.1 Baseline and prime

The descriptive statistics for local antecedent interpretation in prime and baseline conditions are as follows 2. The full score for experimental sentences in each condition was 15, and the mean score of the prime condition was 9.19 (*N* = 36, SD = 2.90, Variance = 8.40), higher than that of the baseline condition of 8.42 (*N* = 36, SD = 3.65, Variance = 13.34). In other words, participants’ accuracy was higher when the experimental sentence was preceded by a prime sentence than when it was preceded by a baseline sentence.

In order to explore whether there was an immediate priming effect more accurately, statistical inference was applied. First, the Shapiro–Wilk test was applied to test the normality of the data. Both scores of experimental sentences following prime sentences and baseline sentences were normally distributed since the two *p*-values fell above the significant level (*p* = 0.52, 0.21). Therefore, a paired sample *t*-test was conducted to compare the number of local interpretation choices preceded by the baseline sentences and by the prime sentences. The analysis revealed a mean difference of 0.778, accompanied by a standard deviation of 2.231 and a standard error mean of 0.372. Moreover, the 95% confidence interval spans from −0.023 to 1.533.. A significant difference was found between the two conditions (*t* = 2.092, *p* = 0.044, df = 35), demonstrating that there were significantly more NP2 choices following the prime sentences than following the baseline sentences. In response to the first research question, there was a comprehension priming effect during reflexive interpretation when no content words were shared across prime and experimental sentences.

### 4.2 Pretest, immediate posttest, and delayed posttest

#### 4.2.1 Analysis of the overall score

Thirty-three out of 36 participants who took part in the first session continued the experiment a week later. In order to test the time course of the priming effects in reflexive comprehension, the scores of 10 experimental sentences in the pretest, immediate posttest, and delayed posttest were compared. The full score of each test was ten. In this part, the two types of experimental sentences varying in pragmatic or semantic bias were analyzed as a whole to attain an overall result. The descriptive statistics of the scores of the three tests are indicated in Table 2. The mean scores at the three time points were 3.639, 5.083, and 3,939, respectively. These intermediate participants did not show a nativelike local interpretation bias in the pretest. The mean score increased in the immediate posttest and then decreased a little in the delayed posttest but was still higher than that in the pretest. Participants’ performance was more heterogeneous in the delayed posttest. In order to verify the statistical significance of the increase from the pretest to the two posttests, a statistical assessment was made. First, the Shapiro–Wilk test showed that only the score of the delayed posttest was normally distributed. As a result, the Wilcoxon signed ranks test, a non-parametric analysis, was performed. Comparisons between the pretest and the immediate posttest and between the pretest and the delayed posttest are presented in Table 3. The results showed a significant increase in NP2 choices in the immediate posttest (*p* = 0.001), while the difference between the pretest and the delayed posttest was not significant (*p* = 0.505). Thus, there was a significant cumulative priming effect after participants finished the whole reading comprehension task, but the priming effects disappeared after a week.

**Table 2:** Descriptive results of scores of pretest, immediate posttest, and delayed posttests.

	Mean	SD	*N*
Pretest	3.6389	1.82291	36
Immediate posttest	5.0833	2.32225	36
Delayed posttest	3.9394	2.58529	33

**Table 3:** Wilcoxon signed ranks tests.

	*Z*	Asymp. Sig.
Immediate posttest-pretest	−3.285	0.001
Delayed posttest-pretest	−0.667	0.505

Table 2 Descriptive results of scores of pretest, immediate posttest, and delayed posttests.

Table 3 Wilcoxon signed ranks tests.

Although comprehension priming was not observed after 1 week based on the analysis of the aggregate data, some of the participants still showed a change in their initial comprehension biases. For example, one-third of the participants (11 out of 33) reached an accuracy of 60% in the delayed posttest, compared with only two in the pretest. The consistency that each individual exhibited was analyzed. The criterion for an individual’s consistency varied in previous studies. It was believed that performance at 100% accuracy was unusual and that a certain amount of “noise” might exist in the data. The criteria for consistency proposed in previous studies included three out of four (Thomas, 1991; Wu et al., 2020), two out of three (Thomas, 1991), and four out of five (Jiang, 2007). This study used the 80% criterion to classify the participants. Specifically, when a participant reached an accuracy level of 80%, the participant systematically accepted the locality constraint. When a participant’s accuracy was 20% or lower, the participant rejected the locality constraint. Other performances would be classified as inconsistent. The classification of participants under the consistency criterion of 80% is shown in Table 4. Only participants who had finished both sessions were included in the analysis. In the pretest, no participant consistently accepted the locality constraint and nine participants (27.3%) consistently rejected it. After the treatment, seven participants (21.2%) were consistently correct, and only three (9.1%) were consistently incorrect. Even in the delayed posttest after 1 week, four participants (12.1%) still reached the accuracy of 80%. Therefore, the individual data showed that some participants were more susceptible to comprehension priming, and they were able to acquire the abstract local-binding rule with repeated exposure to local interpretations.

**Table 4:** Classification of participants under the consistency criterion of 80%.

	True		False		Inconsistence	
	Count	Percentage	Count	Percentage	Count	Percentage
Pretest	0	0	9	27.3%	24	72.7%
Immediate posttest	7	21.2%	3	9.1%	23	69.7%
Delayed posttest	4	12.1%	9	27.3%	20	60.6%

Table 4 Classification of participants under the consistency criterion of 80%.

#### 4.2.2 Analysis of the scores of the biased experimental sentences

In order to explore whether participants have acquired the true locality constraint or whether they can get over the pragmatic pressure, their performances on the biased experimental sentences were examined individually. The participants were considered to be using a true locality constraint only when they resisted pragmatic favoring of the distant NP and bound the reflexive exclusively to the local antecedent. Table 5 displays the scores of biased experimental sentences in three tests. The full score of each test was five. The mean scores for the biased experimental sentences in these three tests were 1.00, 2.00, and 1.36, respectively. The mean score increased from the pretest to the immediate posttest and decreased in the delayed posttest, similar to overall performance. In order to test whether the mean scores varied significantly over time, inferential statistics were presented.

**Table 5:** Descriptive results of neutral and biased experimental sentences at three time points.

Time	*N*	Mean	Std. deviation
Pretest	36	1.0000	0.89443
Immediate posttest	36	2.0000	1.43427
Delayed posttest	33	1.3636	1.36515

Table 5 Descriptive results of neutral and biased experimental sentences at three time points.

The Shapiro–Wilk test showed that none of the scores were normally distributed. A Friedman test was conducted. There were differences across the three time points (*p* = 0.006), and the pairwise comparisons (see Table 6) found that the scores of the immediate posttest were significantly higher than those of the pretest (*p* = 0.02). Therefore, participants showed a cumulative priming effect in reflexive interpretation for the biased experimental sentences. After 1 week, no priming effect was found.

**Table 6:** Pairwise comparisons of scores of biased experimental sentences.

Sample 1-Sample 2	Test statistic	Std. error	Std. test statistic	Sig.	Adj. Sig.^a^
Pretest-delayed posttest	−0.333	0.246	−1.354	0.176	0.527
Pretest-immediate posttest	−0.667	0.246	−2.708	0.007	0.020
Delayed posttest-immediate posttest	0.333	0.246	1.354	0.176	0.527

The significance level is 0.05; ^a^Significance values have been adjusted by the Bonferroni correction for multiple tests.

Table 6 Pairwise comparisons of scores of biased experimental sentences.

Individual data was also analyzed to attain a more accurate result. Participants who chose the local antecedent choices no fewer than four out of five times were considered to have consistent local interpretations (labeled as T). Those who chose the local antecedent choices no more than once were considered to have consistent wrong interpretations (labeled as F). Others were considered inconsistent (labeled as I). The consistency of participants for the biased experimental sentences in the pretest, immediate posttest, and delayed posttest is presented in Table 7. Only the data of the 33 participants were analyzed. The descriptive statistics showed that in the pretest, no participant was consistently correct in comprehending biased experimental sentences. The number of participants who were consistently correct increased after the exposure phase but decreased after 1 week. Three participants had successfully changed their initial interpretation biases and chose the local antecedent consistently in pragmatically unsupportive sentences in the delayed posttest.

**Table 7:** Descriptive results of the consistency of the participants.

Sentence type		Pretest		Immediate posttest		Delayed posttest	
		Count	Percentage	Count	Percentage	Count	Percentage
Biased	T	0	0	7	21.2%	3	9.1%
	F	24	72.7%	18	54.5%	20	60.6%
	I	9	27.3%	8	24.2%	10	30.3%

Table 7 Descriptive results of the consistency of the participants.

A Cochran’s Q test was conducted to explore whether there was a significant difference in the percentage of participants who were consistently correct among the three tests. For biased experimental sentences, the difference at the three time points was statistically significant (*p* = 0.003). Pairwise comparisons were made with the “exact” McNemar’s test. As shown in Table 8, there was only a statistically significant difference between the pretest and the immediate posttest (*p* = 0.008), indicating that the number of participants who were consistently correct in comprehending the biased sentences increased significantly in the immediate posttest compared to the pretest.

**Table 8:** “Exact” McNemar’s test.

	Pretest- immediate posttest	Pretest-delayed posttest	Immediate posttest-delayed posttest
*N*	33	33	33
Exact sig. (two-tailed)	0.016	0.250	0.125
Exact sig. (one-tailed)	0.008	0.125	0.063
Point probability	0.008	0.125	0.063

Table 8 “Exact” McNemar’s test.

## 5 Discussion

### 5.1 The occurrence of the structural priming in L2 reflexive interpretation

Based on the results of this study, it is evident that structural priming effect occurs in comprehending English reflexive pronouns. Furthermore, the observed priming effect persists even in scenarios where there is no repetition of the verb between the priming and experimental sentences. These outcomes align with previous findings by Contemori (2021), Contemori (2023), and support Ledoux et al. (2007) assertion that verb repetition is not a prerequisite for the occurrence of the structural priming effect.

In comparison to Contemori (2023), this study differs in terms of participants’ native languages and target structures. In this study, the participants were native Chinese speakers, and the target structure was constraint rules of English reflexive pronouns. The results indicated a significant improvement in immediate posttest scores compared to pretest scores (*p* = 0.001), demonstrating the occurrence of priming effect and suggesting that learners can acquire knowledge of the constraint rules of English reflexive pronoun through exposure. According to the modeling literature, the participants were Spanish speakers, and the target structure was the English pronoun. The results showed a significant improvement in the learners’ immediate posttest scores compared to their pretest scores (*p* = 0.007), indicating a priming effect. Therefore, the occurrence of structural priming effect is independent of differences in learners’ native languages and target language structures. Priming occurs ubiquitously and the priming mechanism is universal.

Our study confirms that verb repetition is not a prerequisite for the occurrence of priming effect, and supports that source of the comprehension priming relies on generalization that extend beyond verbs. The finding adds to the evidence that EFL learners could develop lexically-independent representations at the early stage of language learning with exposure to only a limited amount of input, or the abstract representation becomes activated after exposure to a certain amount of positive evidence. The EFL learners in this study had not acquired the locality constraint of English reflexive pronouns until they were exposed to 15 prime sentences. They might have activated the co-referential relationship between the reflexive pronoun and the local NP.

### 5.2 The persistence of the structural priming in EFL reflexive interpretation

This study confirmed the persistence of structural priming effects. The structural priming effects were observed immediately after the presentation of prime sentences, cumulatively after the entire priming phase, but disappeared after 1 week. The finding that structural priming could persist over intervening items accorded with previous observations (Bock and Griffin, 2000; Thothathiri and Snedeker, 2008; Contemori, 2021). However, the more long-lasting priming effect observed in previous studies was not found in this experiment. For example, in examining children’s production of passive structure, Branigan and Messenger (2016) found a priming effect that held for 1 week, and Savage et al. (2006) found a more long-lived priming effect that could persist for 1 month. However, Kidd’s study (Kidd, 2012), like the present one, did not find any priming effect after 1 week, and the author explained that the reasons lay in the limited number of prime stimuli and the absence of naturalistic contexts. As in the field of EFL comprehension, some studies have discovered a long-term priming effect that can last for around a week. For example, in the study of Wei and Jin (2020), the Chinese EFL learners showed the comprehension priming effect with object relative clauses a week after the treatment that contained 18 tokens. The EFL learners in the present study only comprehended 15 prime stimuli. Therefore, the relatively short-lived priming effect might also be attributed to the limited number of prime stimuli, following Kidd’s view. However, with the same number of prime stimuli and a similar learning target, that is, the interpretation bias of pronouns in a specific structure, Contemori et al. (2022) observed robust structural priming effects for Spanish English learners even after 6–10 days. In order to reconcile the contradictory findings of this experiment with previous studies, further explanations for the absence of the more long-term priming effect are made.

The first possible reason may lie in the inherent differences in learning targets. The study of Contemori et al. (2022) investigated pronoun interpretation, while the present study focused on reflexive pronouns. The results could be explained by O′Grady’s processing determinism combined with Bjork’s desirable difficulty framework. O’Grady (2015) proposed that reflexive pronouns and plain pronouns manifest different development profiles, with the former characterized by early mastery and the latter by the potential for early confusion. The processing determinism holds that development in language acquisition is not shaped by a principle of Universal Grammar but by two types of processing pressure: internal efficiency-related factors relevant to easing the burden on working memory and external input-related factors such as frequency, with the former playing a more important role. According to this viewpoint, English reflexive pronouns are acquired earlier because the processing cost is reduced when the reflexive pronouns are interpreted locally, whereas more processing cost is required when searching for the plain pronoun’s antecedent in the discourse. Therefore, comprehending pronouns is more costly in processing and more difficult than comprehending reflexive pronouns. According to the desirable difficulty framework proposed in the domain of cognitive psychology (Bjork, 2018), procedures posing certain difficulties and appearing to slow the learning rate often enhance long-term retention and transfer of to-be-learned skills and knowledge. Given that comprehending pronouns is more challenging and complex, pronouns are processed more deeply and therefore benefit more from the comprehension priming task, and the retention of pronoun interpretation preference is consequently more long-lived.

The second reason for the absence of the more long-term priming effect might be attributed to the testing materials used in the immediate posttest. The immediate posttest consisted of only filler sentences and experimental sentences, and the experimental sentences were still ambiguous for those who had not fully learned the local-binding rule during the exposure phase. Half of the experimental sentences were set in pragmatically unsupportive contexts in which the distant antecedent seemed more plausible. This type of experimental sentence might play a similar role as the prime stimuli and reinforce the distant binding interpretation and incorrect connections between structure elements. For those participants who had not systematically acquired the abstract local-binding rule, they might assume that reflexive pronouns in these biased experimental sentences were indexed to the distant NP1. These misleading experimental sentences in the immediate posttest would encourage the participants to make unfavorable adjustments to the probability distribution. Therefore, the immediate posttest might have a negative effect on the subsequent comprehension in the delayed posttest. For participants who had already formed abstract representations and acquired the abstract local-binding rule in the exposure phase, they were less likely to be influenced by the semantic or pragmatic biases and would insist on binding the reflexive to the local antecedent in the delayed posttest.

### 5.3 The mechanism underlying structural priming in comprehension

Based on whether the priming is transient or long-lasting, the results could provide some insight into mechanism underpins comprehension priming.

First, according to the residual activation model, the lexical-independent priming effect is attributed to the activation of the combinatorial nodes, and the lexical boost, that is, the stronger priming effect when there is an overlapping verb, is due to the activation of both the combinatorial node and the link between the combinatorial node and the primed verb. Since activation is pretty transient in nature, the priming effects should not persist across many (if any) intervening structures. Studies that favor this account often observe a priming effect in processing the adjacent sentence without inserting any intervening sentences (Pickering and Branigan, 1998). Therefore, the immediate priming effect observed in the present study could be explained by activation. The prime and experimental sentences shared the same structure, and the prime sentence described a self-generated event. There was a possibility that the concept got activated and affected learners’ comprehension of the target sentence. In addition, though there was no verb repetition across the prime and target sentences, both sentences contained a reflexive. The learners might activate a link between the reflexive and the local antecedent interpretation in prime sentences, and were more likely to comprehend in this way in subsequent reading. However, studies that oppose the residual activation account have found that abstract priming effects can last for two or more intervening utterances. Similar to the findings of Contemori et al. (2022), this study also observed a cumulative priming effect after the whole exposure phase. The immediate posttest did not contain any prime sentences, and there were at least three filler sentences between the priming phase and the test. Therefore, the time course of the priming effect was relatively long, which was in contradiction with the prediction of the residual activation account. Although this model could account for the immediate priming effects, it could not account for the long-term cumulative priming effect.

The second account, namely, the implicit learning account, could explain both short-term and long-term priming effects. In the framework of this account, priming could be considered as a type of implicit learning that can last for a long time. The EFL learners were expected to learn the abstract local-binding rule, and the rule was not explicitly taught. The experiment was designed as an English reading comprehension test to prevent the participants from being aware of the purpose of the study. During the comprehension of the prime sentences where the reflexive pronouns were always bound to the local antecedents, participants unconsciously strengthened the connections between the elements of the target structure and formed an abstract generalization that the reflexive was always co-indexed with the subject of the subordinate clause in bi-clausal finite sentences. The priming effect not only affected immediate referent choices in the priming phase but also persisted undiminished after several intervening filler sentences in the immediate posttest. Some individuals even consistently selected the local antecedent choices in the delayed posttest. Therefore, the observed priming effects were, at least in part, a form of implicit learning instead of short-term activation. However, it is unclear whether the immediate priming and the cumulative priming were driven by the same mechanism. These observed priming effects might be driven by the single implicit learning mechanism or be driven by different mechanisms under the dual mechanism account. But if both mechanisms work, the dual mechanism account could explain the stronger priming effect in the immediate posttest and its absence after 1 week.

In conclusion, the findings of this study lend support to the hypothesis that comprehension priming occurs via an implicit learning mechanism. The activation account could explain the immediate priming effect, but not all of the results.

## 6 Conclusion

This study examined the effectiveness of structural priming in changing EFL learners’ interpretation bias to approximate the nativelike pattern. The three research questions were answered based on the results of a battery of pencil-and-paper comprehension tests. First, structural priming occurred when Chinese EFL learners comprehended reflexive pronouns embedded in bi-clausal finite sentences. Second, the priming effects were found immediately after the presentation of prime stimuli and after the whole exposure phase, but not after 1 week. The absence of the long-term priming effect after 1 week was incompatible with the study of Contemori et al. (2022). The possible reasons might be attributed to differences in target structures, testing materials, and individual internal factors. Third, the accuracy of comprehension for the biased experimental sentences increased significantly. The results suggested that reflexive pronouns’ locality constraint could be fully acquired through repeated exposure.

Two aspects of implications can be derived from this study. First, from a theoretical perspective, the study sheds light on a mechanistic explanation for the structural priming effect in comprehension, indicating that an implicit learning mechanism is a more probable underlying mechanism. Second, from a practical perspective, the results have implications for teaching. This study provides evidence that contextualized input has a positive effect on learning abstract language rules. When designing teaching content, other enhancement techniques that contribute to the formation of nativelike patterns can be employed.

Additionally, several limitations need to be acknowledged for the future study. First, the materials need to be optimized. For example, some of the sentences were from previous publications, while others were created by imitating these sentences. There might be differences between the materials used in this study and the natural discourse. Second, the conclusion of this study lacks representativeness and variety. Evidence from more participants on more sentence patterns is needed to draw a safer conclusion.

This study also points out several future directions that await further investigation. First, in order to verify the assumption proposed in this study, more priming studies are needed to investigate reflexive interpretation in more sentence structures that are less common and more difficult. In this study, both types of priming effects may interact or operate simultaneously, because both structure and discourse-level information can contribute to processing and interpretation. Future investigation can examine the interplay of structural and discourse priming effects in language comprehension, that is, to compare priming effect in two conditions: one integrating both structural and discourse priming, and the other concentrating solely on one type of priming. Second, structural priming in comprehension might be influenced by many other factors that have not received enough attention. Future research should look into the effects of these factors as well as the interactions between them. Third, future studies can use more types of measures such as reading times (RTs) and eye movement in an online self-paced reading task.

## Data Availability

The raw data supporting the conclusions of this article will be made available by the authors, without undue reservation.
